# Iron Homeostasis Disruption and Oxidative Stress in Preterm Newborns

**DOI:** 10.3390/nu12061554

**Published:** 2020-05-27

**Authors:** Genny Raffaeli, Francesca Manzoni, Valeria Cortesi, Giacomo Cavallaro, Fabio Mosca, Stefano Ghirardello

**Affiliations:** 1 Fondazione IRCCS Ca’ Granda Ospedale Maggiore Policlinico, NICU, 20122 Milano, Italy; genny.raffaeli@unimi.it (G.R.); francescamanzoni.unimi@gmail.com (F.M.); valeria.cortesi92@gmail.com (V.C.); giacomo.cavallaro@policlinico.mi.it (G.C.); fabio.mosca@unimi.it (F.M.); 2 Department of Clinical Sciences and Community Health, Università degli Studi di Milano, 20122 Milano, Italy

**Keywords:** iron, redox unbalance, prematurity, transfusion, anemia, blood-sparing

## Abstract

Iron is an essential micronutrient for early development, being involved in several cellular processes and playing a significant role in neurodevelopment. Prematurity may impact on iron homeostasis in different ways. On the one hand, more than half of preterm infants develop iron deficiency (ID)/ID anemia (IDA), due to the shorter duration of pregnancy, early postnatal growth, insufficient erythropoiesis, and phlebotomy losses. On the other hand, the sickest patients are exposed to erythrocytes transfusions, increasing the risk of iron overload under conditions of impaired antioxidant capacity. Prevention of iron shortage through placental transfusion, blood-sparing practices for laboratory assessments, and iron supplementation is the first frontier in the management of anemia in preterm infants. The American Academy of Pediatrics recommends the administration of 2 mg/kg/day of oral elemental iron to human milk-fed preterm infants from one month of age to prevent ID. To date, there is no consensus on the type of iron preparations, dosages, or starting time of administration to meet optimal cost-efficacy and safety measures. We will identify the main determinants of iron homeostasis in premature infants, elaborate on iron-mediated redox unbalance, and highlight areas for further research to tailor the management of iron metabolism.

## 1. Iron Homeostasis and Prematurity

Iron is an essential micronutrient that plays a pivotal role in early development, being involved in hemoglobin synthesis, oxygen delivery, electron transfer, energy metabolism, and cell differentiation [1]. Nowadays, there is consistent evidence about the relationship between in utero iron supply and subsequent cognitive and neurobehavioral outcomes [2,3]. Iron homeostasis is a balance between iron absorption, storage, and recycle by erythroid precursors. The total body iron is distributed into three compartments [4]. The majority of total body iron (at least two-thirds) is within erythrocytes, in the form of hemoglobin (Hb). A small part (about 15%) is “storage iron”, mostly kept within ferritin or hemosiderin in liver and spleen, ready to be mobilized [5,6,7]; the remainder (10%) is non-heme-non-storage tissue-circulating iron. The latter is bound to serum transferrin, an iron chelator that keeps iron in a soluble, inert, reduced state, preventing toxic oxidative reactions [7]. Hepcidin is a peptide hormone acting as a negative feedback regulator of iron metabolism, by modulating the expression of ferroportin, which is an iron-exporter transmembrane protein of enterocytes and macrophages. Hepcidin synthesis in the hepatocytes is determined by circulating and stored iron, inflammation, and erythropoietin. In the event of high iron levels or inflammation, hepatic hepcidin release is increased and ferroportin expression is downregulated. Conversely, anemia, hypoxia, and low iron levels are associated with reduced hepcidin expression, leading to the increased activity of ferroportin and the mobilization of iron reserves [4,6]. Iron deficiency (ID) is a relevant public health problem and is the most common single-element deficiency worldwide, affecting around 2 billion people globally [1,8]. Both pre-existing anemia and increased iron requirements during pregnancy make pregnant women particularly vulnerable to develop iron deficiency anemia (IDA) [2]. It is estimated that maternal IDA affects approximately 30–50% of pregnant women in developing countries and less than 1% in developed countries where iron supplementation is part of routine care [9]. There are no conclusive results about the relationship between maternal and neonatal iron status. Past studies showed that early maternal sideropenic anemia doubles the risk of preterm delivery and low birth weight [10,11]. Iron endowment at birth depends on iron stored during gestation, tightly connected to maternal iron status, and iron received perinatally as Hb, depending on the time of cord clamping [12]. Adequate iron stores at birth are necessary to satisfy requirements in the first 6–9 months of age, when the neonatal gut is not fully developed to properly regulate iron intake and breast milk cannot meet the recommended needs [2]. Poor levels of iron at birth, on the contrary, predict future ID and/or IDA during infancy [12]. 

In the same way, iron homeostasis can be deeply influenced by prematurity. Iron is mostly (>66%) transferred during the third trimester of pregnancy [10] so that total iron stores are inversely related to gestational age. Moreover, many pregnancy complications, such as multiple pregnancies, obesity, gestational diabetes, and hypertension with intrauterine growth restriction (IUGR) [13], can also induce impaired iron endowment due to chronic placental insufficiency [10,14]. Iron demand, as with all nutrients, increases during fetal and neonatal development [15]. In premature infants, the early onset of erythropoiesis and the fast catch-up growth occurring in the first 6–8 weeks of life require additional iron, especially in the most immature neonates [16]. Moreover, premature infants’ iron stores can be further depleted by recurrent phlebotomy and administration of recombinant human erythropoietin (rHuEPO) in the absence of adequate iron supplementation.

Low iron reserves and increased iron requirement explain why preterm infants are at high risk of ID/IDA and need iron supplementation [1,14]. It is estimated that between 25% and 85% of premature newborns develop iron deficiency, usually in the first six months of life [16]. In preterm neonates, ID can affect the majority of organs, causing poor growth, temperature instability, thyroid dysfunction, decreased cell-mediated immune response and impaired DNA and collagen synthesis, even before microcytic and hypochromic anemia appears [16,17]. However, the main concern is the impact of ID on brain development [9,18].

On the other hand, premature infants are vulnerable to oxidative stress caused by non-transferrin-bound iron (NTBI) overload due to the immature antioxidant system and the high transferrin saturation [1,6,19,20]. NTBI can derive from high doses of oral or parental iron supplementation and recurrent erythrocyte transfusions [14]. The latter is relevant if we consider that around 40% of very low birth weight (VLBW) and more than 90% of extremely low birth weight (ELBW) infants receive at least one blood transfusion during hospitalization [21]. Birth, itself, is an oxidative challenge since there is a rapid increase in the oxygen concentrations compared to the hypoxic intrauterine environment [22]. Indeed, plasma NTBI concentrations appear to be higher in the neonatal period if compared to the following ages [19,20]. Iron excess cannot be removed by physiological pathways and can accumulate and generate free radicals resulting in cellular toxicity. The same toxic reaction can be produced when the iron is delocalized from its binding protein, as it happens in the setting of hypoxia [6,23]. As a result, the sickest premature neonates are prone to develop complications that may share the same etiology, grouped under the expression coined by Saugstad, “the oxygen radical disease of neonatology” [24]: bronchopulmonary dysplasia (BPD), retinopathy of prematurity (ROP), necrotizing enterocolitis (NEC), intraventricular hemorrhage (IVH), periventricular leukomalacia (PVL), and punctate white matter lesions (PWM) [1,21,25].

## 2. Iron and Brain Development

Iron is critical for brain development in the fetal and early neonatal period, and major issues are related to the long-lasting effect of ID on neurodevelopment. Biologically, in the case of negative iron balance, iron is redistributed following a hierarchical strategy and primarily used by red cells to the detriment of other tissues: firstly liver, followed by the heart, skeletal muscle and. finally. brain [4,26]. It results that ID can injure the brain even in the absence of IDA [9,27,28]. Indeed, ID can impact brain functions because several iron-dependent enzymes are essential for neurotransmitter synthesis, myelination, synaptogenesis, gene expression and neuronal energy production [4,26].

The American Academy of Pediatrics (AAP) has highlighted the relevance of ID and IDA screening during infancy [2], due to both short- and long-term negative consequences of IDA on motor, cognitive, social, and behavioral development [3,29,30].

Both timing and duration of ID are critical because the brain’s affected areas are in a crucial developing phase at the time of ID [9]. In particular, ID during gestation and lactation carries the most severe effects, as it occurs during the brain’s growth spurt, [9]. Rodent models have shown that early (from the late fetal period to 24 months of postnatal age) ID can affect the dopaminergic system in the striatum, and alter gene expression and dendritogenesis in the hippocampus, leading to motor and memory life-long alterations, respectively [9]. In the same way, the composition of myelin lipids persists irreversibly altered in adulthood despite iron treatment [9].

Intrauterine ID, as well, can have life-long consequences as proved by the association between cord ferritin levels below the lowest quartile (<76 mg/L), a marker of in-utero fetal iron status [31], and language and fine motor skills delay at five years of age [2,32].

Scarce data evaluated the relationship between in-utero ID and long-term neurobehavioral effects in preterm infants. Premature infants suffering from fetal ID have abnormal auditory brainstem-evoked responses (ABR), a marker of brain maturation, with longer latencies in the perinatal period [31]. Moreover, anemic preterm infants show altered neurological reflexes at 37 weeks, probably suggesting impaired myelination or neurotransmitters synthesis [33]. Interestingly, ID in premature infants tends to express through motor deficits, while cognitive impairment prevails in term infants [16].

Based on preclinical evidence [34], limited benefits were reported for the promotion of mental or motor development in infants with IDA [35,36]. This result could be explained by the late timing of the onset of supplementation: ID begins prenatally, and interventions targeting infancy or early childhood were too late [37]. On the other hand, various studies showed that iron supplementation in infants at risk of ID had positive effects on motor and cognitive development [38,39,40]. Similarly, early (<61 days of life) iron supplementation in premature infants improves neurocognitive outcome VLBW neonates [41].

The main core of the debate is related to the fact that not only ID can be troublesome, but also iron excess, as mentioned above, may induce oxidative stress, which contributes to the pathophysiology of several prematurity-related diseases [25].

Indeed, preterm infants often experience repeated episodes of hyperoxia, hypoxia, and ischemia resulting in free radicals and reactive oxygen species (ROS) production that these patients cannot cope with, due to an ineffective anti-oxidant system [25]. Specifically, several antioxidant enzymes, such as the superoxide dismutase, catalase, and glutathione peroxidase, show a decreased activity in the immature brain [42].

Iron becomes toxic when not bound to proteins [43,44]. Neonates, particularly if born prematurely, are prone to generate NTBI as a result of repeated episodes of hypoxia, acidosis, and ischemia in the perinatal period, when plasma transferrin, ceruloplasmin and total iron bind capacity (TIBC) are constitutionally low [42]. Indeed, amoeboid microglial cells of the periventricular white matter show increased intracellular iron concentration after a hypoxic insult that can lead to oligodendrocyte cell death and axonal swelling [45].

Growing evidence suggests a role for iron in hypoxic-ischemic encephalopathy (HIE). Indeed, high amounts of NTBI were found in the cerebrospinal fluid (CSF) and the serum of newborns after a hypoxic-ischemic injury [23,46]. NTBI concentrations are directly related to the severity of brain injury [23], and blood NTBI has been considered an early predictive marker of long-term neurodevelopmental outcomes [20,46]. Iron-mediated radical factors disrupt the blood-brain barrier and cause endothelial necrosis following a hypoxic-ischemic injury [42].

**Figure 1:**
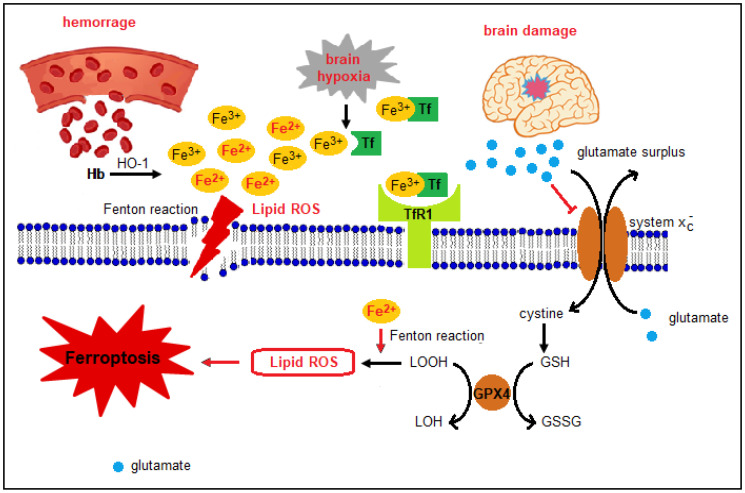
Presumptive molecular pathways of ferroptosis following brain injury in the developing brain. Excess free iron in the brain may be the result of Hb degradation by HO-1 after intracerebral hemorrhage. Similarly, a hypoxic-ischemic insult enhances iron liberation from its binding proteins. Fe^2+^, the reactive form of iron, promotes ROS production via the Fenton reaction leading to lipid peroxidation and membrane damage while the damaged brain releases glutamate. High extracellular glutamate concentrations inhibit the cystine/glutamate antiporter system xc- thus reducing cellular cystine levels, necessary for GSH synthesis. Reduced intracellular cystine concentration indirectly inactivates GPX4, the enzyme responsible for lipid hydroperoxide reduction and GSH consumption. The accumulation of lipid hydroperoxides in an enriched Fe^2+^ environment leads to significant lipid ROS formation that induces membrane permeabilization and ferroptosis [6,47]. Fe^2+^: ferrous cation; GPX4: glutathione peroxidase 4; GSSG: oxidized GSH; GSH: reduced glutathione; Hb: hemoglobin; HO-1: heme oxygenase 1; LOOH: lipid hydroperoxides; LOH: lipid alcohols; ROS: reactive oxygen species; TF: transferrin; TfR1: transferrin receptor 1. Adapted from Wang et al. [6].

Similarly, iron-induced neuronal toxicity is a determinant of IVH pathophysiology in the preterm brain as the free iron released from heme destruction after intracerebral hemorrhage (ICH) contributes to secondary brain injury and post-hemorrhagic ventricular dilatation [43]. Indeed, while the primary hit lies in the presence of the hematoma itself, the secondary injury refers to the subsequent release of neurotoxic iron-related compounds from the hematoma. Specifically, heme oxygenase catabolizes heme into carbon monoxide, biliverdin, and free iron. The free iron accumulation increases the risk of oxidative damage to lipids, protein and DNA, by inducing the free radical production by means of the Fenton reaction [43]. In both HIE and IVH, the potential mechanism by which iron can damage the neonatal brain has been recently studied and has been named “ferroptosis”, a non-apoptotic iron-dependent pathway of cell death (Figure 1) [43].

NTBI can trigger ferroptosis, inducing a process mediated by lipid peroxidation and glutathione consumption with subsequent hydroxyl radical production via Fenton and Haber–Weiss reactions. Free radicals and nitric oxide generated during brain reperfusion following hypoxic events activate a series of chain reactions that mobilize an increasing amount of iron from its binding proteins and red cells [48], thus amplifying cell death and brain injury [6]. To support this theory, high concentrations of malondialdehyde, a product of lipid peroxidation, have been found in CSF of infants with HIE [23]. This pathogenetic mechanism is relevant in the neonatal brain if we consider the large amount of iron and polyunsaturated fatty acids that constitute the lipidic membrane in the white matter, readily susceptible to free radical attack [46]. Additionally, CSF is characterized by a low TIBC that can bind NTBI and an unbalanced relationship between ceruloplasmin and vitamin C that favors the latter and results in iron oxidation in the active ferrous form [20,49]. Even orally-administered iron can damage brain: preclinical studies demonstrated Parkinson-like neurodegeneration in adults that have been fed with great amounts of enteral iron during breastfeeding [16].

Iron chelators have shown a neuroprotective effect in animal studies [23]. rHuEPO likewise protects against inflammation, apoptosis, and oxidation and decreases unbound iron by stimulating erythropoiesis [43]. In preclinical models of neonatal brain damage, rHuEPO acts as a neuroprotector, promoting neurogenesis and neural regeneration after an insult [50]. In clinical practice, rHuEPO is administered at the dose of 300–500 U/kg for the prevention and treatment of the anemia of prematurity [51]. Low-doses of rHuEPO has been previously associated with better short-term and long-term neurological outcomes in both full-term and preterm infants [50,51]. However, the results from a recent randomized trial, enrolling extremely preterm infants to receive high-dose of rHuEPO (1000 U/kg) in the first weeks of life, did not improve neurodevelopmental at two years of age [52], if compared to the placebo group. 

## 3. Iron Status Measurement

Even if bone marrow aspiration is considered the gold standard for ID diagnosis, it has been replaced in clinical practice by other less invasive laboratory parameters [53], classified in hematological and non-hematological tests. The former includes Hb, mean corpuscular volume (MCV), reticulocyte count, and hemoglobin reticulocytes content, designated as the mean cellular hemoglobin content of reticulocytes (CHr) or reticulocyte hemoglobin equivalent (RET-He). The latter include serum ferritin (SF), transferrin saturation, soluble transferrin receptor (STfR1), zinc protoporphyrin to heme ratio (ZnPP/H).

The changes in iron status tests in response to ID, IDA, or iron overload are reported in Table 1. [14,54,55].

The assessment of neonatal iron status is a challenging task as neonatal blood sampling requires a well-trained phlebotomist and is not routinely performed among healthy newborns. For this reason, neonatologists, instead of “normal values” established from healthy neonates, use “reference ranges” that include values between the 5th and the 95th percentile derived from newborns with minor pathology [56].

Moreover, most hematological parameters are influenced by gestational age and postnatal developmental changes [56]. As a result, gestational age-specific laboratory markers are needed. The site of blood collection (venous, arterial, capillary) and the use of different reagents can further impact on values [57]. Reference ranges for the main iron status parameters in term and preterm neonates are listed in Table 2.

Given the relevance of iron homeostasis in neonates, especially among premature ones, and the potential reversibility of pathologic conditions with prompt treatment, the reliable measurement of iron status is pivotal [57]. However, normative values of the main parameters used for the diagnosis of ID and IDA in premature infants are still lacking [62].

The iron reduction can be summarized through three stages of increasing severity:

1. Iron depletion: decrease of total iron storage, as reflected by SF, the first laboratory index to decline in ID;
2. Iron deficient erythropoiesis: initial reduction of Hb concentration due to complete depletion of iron stores; and
3. Iron deficiency anemia: ID associated with anemia, defined by WHO as Hb level two standard deviations below the median of a healthy population of the same age and sex [5,14].

Hb is the most frequently used hematologic parameter to screen for ID in infants. In clinical practice, the terms “anemia” and “IDA” have been interchangeably used as if the ID is the only cause of anemia [14,63]. However, Hb alone lacks sensibility and specificity since low Hb levels can derive from several conditions other than ID, such as hemolysis, chronic infections, genetic disease, or other less common nutrient deficiencies, particularly folate or vitamin B12 [14]. For this reason, to establish IDA, Hb measurement should be associated with: (1) SF and C reactive protein (CRP), or (2) CHr, based on AAP recommendations [14].

However, anemia suggests a severe depletion of iron stores. Indeed Hb is a late marker of ID and is not a reliable indicator of neonatal iron status, especially if measured in newborns suffering from chronic hypoxia, where stored iron is prioritized to preserve erythropoiesis to the detriment of other tissues [13].

Since ID alone, even without anemia, may impair neurodevelopment, prompt diagnosis and treatment of ID are essential. The AAP suggests to screen ID through the measurements of (1) SF and CRP, or (2) CHr [14].

Serum ferritin estimates total iron body stores; its cord blood concentration steadily increases throughout gestation [62]. Recently, ferritin concentration has been measured in cord blood in neonates from 23 to 41 gestational age [28]. Specifically, SF values below 35 µg/L indicate ID in preterm infants and correlates with complete depletion of liver iron stores [28]. On the other hand, SF concentrations >300 µg/L depict iron overload [64]. However, SF is an acute phase reactant and can reach high concentration during inflammation and infection [2]. As an increase in SF during co-existing inflammatory processes can mask ID, the AAP suggests the simultaneous measurement of SF and CRP [2]:

- low SF concentrations regardless of CRP level confirm ID;
- increased or normal SF levels associated with normal CRP concentrations rule out ID; and
- when high or normal SF concentrations are associated with increased CRP levels, iron status cannot be assessed [14].

Serum ferritin concentrations increase in the early postnatal period due to hemolysis and delivery itself, so that cord ferritin levels are approximately 1/3 of those in the first 72 h of life [58]. The opposite happens to serum iron concentration, increased in the umbilical vein, as a result of iron transport from the mother to the fetus [58].

RET-He and CHr quantify iron concentration inside reticulocytes and, therefore, by providing a real-time assessment of bone marrow iron status, may represent a preventive screening test for ID [63]. Indeed, their detection may anticipate ID diagnosis, if compared to Hb, which is a late marker of ID, since Hb evaluates the whole red blood cell population. Recent studies suggest a role for CHr in predicting future IDA even among infants; however, its measurement is not readily available in a laboratory setting [63]. A linear correlation has been reported between RET-He and CHr [65].

Similarly, STfR1 concentration is related to intracellular iron stores. High STfR1 concentrations are found in term and preterm neonates as a result of maternal ID, reflecting a poor iron endowment [28]. They are not directly influenced by gestational age [58]. Neonatal reference ranges should be established to improve its use in clinical practice.

ZnPP/H detects zinc incorporation into protoporphyrin IX in erythrocytes [66]. In case of insufficient iron delivery to bone marrow and reduced erythropoiesis [62], iron is replaced by zinc, thus increasing ZnPP/H ratio. Nevertheless, it cannot distinguish whether it is caused by body iron stores depletion or enhanced rate of erythropoiesis, as it happens in premature infants during the first weeks of life. ZnPP/H ratio is inversely correlated with gestational age [59,62]. Higher ZnPP/H ratios are detected in those born to mothers affected by gestational diabetes and IUGR or with chorioamnionitis, suggesting a potential association with inflammatory or infectious processes [28,59,62].

Brain iron concentrations are hardly measurable and can be quantified only at autopsy. Differently, serum ferritin can be used as an indirect index of brain ID while cord ferritin as a marker of in-utero fetal iron status [31]. Neurobehavioral tests are used to investigate multiple brain functions that can indicate the injured brain area, such as the Bayley Scales for Infant Development, Griffith Development Scale, Wechsler Preschool and Primary Scale of Intelligence (WPPSI), and the Wechsler Intelligence Scale for Children (WISC). Nevertheless, they show a low specificity for ID, since other nutrients deficiencies (e.g., zinc, copper, iodine) can lead to similar neurobehavioral abnormalities [6].

## 4. Iron Deficiency and Supplementation

### 4.1. Risk Factors

#### 4.1.1. Maternal Iron Status

In the past, it has been assumed that a poor maternal iron status during pregnancy, unless determining severe IDA, does not affect fetal or neonatal iron endowment, as testified by the absence of association between maternal and cord blood Hb level [11]. Placental transferrin receptors increase in the case of maternal ID to transfer more iron to the fetus [11]. Conversely, it is known that maternal IDA is associated with preterm delivery and low birth weight, both of which lead to decreased neonatal iron stores [11].

However, a direct relationship between cord blood ferritin concentration, maternal Hb, and SF concentration has been found [11]. These observations may indicate that, even if cord blood Hb concentrations are within normal values, those born from mother with ID have reduced iron stores and are more likely to be anemic during infancy [67].

Iron supplementation during pregnancy is beneficial for both the mothers and their infants. The improvement of maternal iron status, even in women with satisfactory iron stores, prevents ID in the subsequent pregnancy [11] and reduces maternal fatigue with a positive impact on mental health, thus producing indirect postnatal benefits on neonatal development [37]. Moreover, iron intake is associated with higher blood ferritin concentration and better neurodevelopmental outcomes in their infants [27].

#### 4.1.2. Maternal Comorbidities

Up to 10% of pregnancies in the developed countries are complicated by IUGR, secondary to placental insufficiency, and structural abnormalities of the placental vessels. These conditions may impair iron transport to the fetus, while chronic fetal hypoxia induces erythropoietin synthesis and subsequent iron use for Hb production [13]. Low cord blood ferritin has been found in about 50% of IUGR infants (<60 ng/mL). Similarly, maternal diabetes mellitus increases fetal metabolism and oxygen consumption by approximately 30% [62]. Hypoxia stimulates Hb synthesis, which requires 3.47 mg of iron for 1 g of Hb [4]. This increased demand depletes heart, liver, and brain iron stores with a severity that is inversely related to maternal glycemic control [4]. Furthermore, the physiological regulatory mechanism of the placenta seems to be lost. Despite fetal hypoxia, placental transferrin receptor (TfR1) concentration is low, indicating a decreased receptor response capacity [68]. 

As a result, IUGR infants and those born to a diabetic mother are at higher risk of brain ID. At autopsy, the most severe cases showed total iron brain content reduced by 30–40%, and more than half of them have low ferritin in cord blood [9].

#### 4.1.3. Prematurity

Around 25–85% of premature newborns develop ID, associated or not with IDA, during infancy [16], usually with earlier onset than in full-term neonates. Indeed, at birth, total body iron concentration is, on average, 75 mg/kg in term neonates versus 64 mg/kg in preterm neonates [66]. Similarly, in the latter group, serum iron concentrations and cord SF are lower with higher sTfR1 levels at birth, when compared to term neonates [62].

Many factors contribute to the negative iron imbalance and can explain why premature infants are so vulnerable to ID and IDA. Firstly, even if placental iron transfer begins in the first trimester of pregnancy, approximately 80% of iron accumulates during the last one [68]. Therefore, the more premature the infant is, the poorer its iron status will be. Postnatally, low iron stores are further reduced by the rapid “catch up” growth with rapid blood volume expansion and increased Hb demand, which requires further iron [62]. Moreover, recurrent phlebotomies for diagnostic purposes cause an extra iron loss in VLBW neonates equal to 6 mg/kg/week, on average [64].

In the past, additional iron was supplied with red blood cells (RBC) transfusions to prevent the anemia of prematurity. In the last years, more restrictive RBC transfusion policies are encouraged, particularly among the sickest extremely preterm neonates, because of the associations between early exposure to RBC transfusions, and increased mortality and short-term morbidities [21,69,70,71].

#### 4.1.4. Low Birth Weight

SGA neonates show lower iron stores when compared to gestation-matched appropriate-for-gestational-age (AGA) neonates in cord blood and at four weeks postnatally [72].

#### 4.1.5. Gender

Gender may influence iron endowment at birth: males have a smaller iron supply at birth reflected by a significantly lower concentration of Hb, MCV, SF, and higher ZnPP and sTfR1 levels at four, six, and nine months when compared with females [73]. These features place male infants at increased risk of ID during infancy. Therefore, to exclude physiological sex-related differences, the construction of gender-specific reference intervals may be beneficial.

#### 4.1.6. Breastfeeding

As human breast milk contains a very low quantity of iron (0.2–0.4 mg/L), exclusively breastfed infants are prone to develop ID [26,74]. The incidence of ID in the first six months of life is 6–15% and 12–37% in industrialized and developing countries, respectively [68]. The iron content in human milk satisfies full-term neonates’ iron demand in the first 4–6 months of life, whereas iron stores of preterm neonates are depleted within 1–4 months after birth [16]. Despite the low iron content in human milk, its bioavailability is around 50%, much higher than in formula milk. Indeed lactoferrin, which is an iron-binding protein contained in breast milk, facilitates iron absorption. In contrast, casein and other cow milk’s proteins in cow milk-based formula have an inhibitory effect on iron absorption [26].

### 4.2. Prevention

Recently, placental transfusion techniques, such as delayed cord clamping (DCC) and umbilical cord milking (UCM), have been implemented in routine neonatal care. DCC consists of delaying umbilical cord clamping for at least 30–60 s after delivery in both term and preterm infants not requiring immediate resuscitation [75]. This practice allows the transfer of 25–35 mL/kg of placental blood to the newborn, thus increasing iron stores by approximately 30% after three minutes of DCC [26]. Indeed, DCC, compared to immediate cord clamping, is associated with higher hemoglobin concentration in first weeks after birth in both term and preterm newborns, and lower rates of RBC transfusions in preterm newborns [75,76,77]. In the long term, DCC maintains its benefits, as evidenced by the higher ferritin levels and the lower incidence of ID at six months of age in term neonates [2,75,78]. Indeed, the amount of blood transferred via DCC is crucial in defining iron endowment at birth [12]. As largely illustrated above, ID and brain development are closely linked. DCC has been associated with improved neurodevelopmental outcome at four years in full-term infants and two years in very preterm newborns [79,80].

UCM consists of a gentle squeezing of the umbilical cord towards the baby two to five times before clamping. Similarly to DCC, UCM enhances iron stores in the first weeks after birth in premature infants as compared to early cord clamping [81]. However, results from a recent randomized clinical trial comparing UCM vs. DCC in preterm infants born before 32 weeks’ gestation have shown a significantly higher rate of severe IVH in the UCM group [82].

Phlebotomy is the first cause of anemia in the first weeks of life [83], especially in the most premature newborns. Therefore, there is increasing attention to minimize blood loss in these patients. To this goal, the use of low blood volume point-of-care testing, non-invasive monitoring and the reduction of unnecessary blood sampling are mainstays of prevention [84]. 

Furthermore, otherwise discarded placental blood has been endorsed as an alternative source for Neonatal Intensive Care Unit (NICU) admission laboratory tests (complete blood count, blood culture, blood type, antibody screen, and metabolic screening) [85,86]. Of note, umbilical cord blood seems not suitable to assess neonatal hemostatic profile, as placental specimens show a procoagulant imbalance if compared to the neonatal counterparts [87]. Drawing blood directly from the umbilical vessels avoids invasive and painful neonatal procedures and results, especially in VLBW infants, in higher hemoglobin concentration, lower rates of RBC transfusions, and need for vasopressors in the first week of life [88]. Additionally, it appears to be a complementary procedure to DCC to maximize circulating blood volume.

### 4.3. Supplementation

#### 4.3.1. Enteral Iron Supplementation

Enteral supplementation is the preferred route of iron administration. It can be provided through iron fortified-human milk, iron-fortified formula, or medicinal elemental iron, in the form of ferrous sulfate or ferrous fumarate. The former has been associated with better absorption, while the latter produced less oxidative stress in vitro. To our knowledge, no studies have compared the two preparations in premature infants [89].

Iron gut absorption has a high inter-individual variability ranging from 10% to 50% of the dose administered and is increased when given with breast milk or with vitamin C [16,57]. Iron intake depends on:

1. Local factors, such as gastric pH and intestinal mucosal function; and
2. General drivers, such as the source of enteral iron (human or formula milk) and the iron stores of the infant [90].

It has been hypothesized that iron absorption and release may be impaired due to enterocytes’ immaturity, thus explaining inadequate iron intakes in orally supplemented neonates [91].

The position paper by the European Society for Pediatric Gastroenterology, Hepatology, and Nutrition (ESPGHAN) recommend an elemental iron intake of 1–2 mg/kg/day for all premature infants with a birth weight less than 2500 g and of 2–3 mg/kg/day for those weighing less than 2000 g [92]. Similarly, the Committee on Nutrition of the AAP suggests a daily iron supplementation of 2 mg/kg for all breastfed preterm infants, from 1 to 12 months of age [93]. An enteral iron dosage >5 mg/kg/day should be avoided in these patients [94].

Iron content in infant formula is 14.6 mg/L and 12 mg/L in standard preterm and term formula, respectively. Based on a standard daily milk intake of 150 mL/kg, formula-fed neonates receive around 1.8–2.2 mg/kg/day of iron [14]. Although being fed with iron-enriched formula, up to 14% of preterm infants develop ID in the first year of life. Thus, iron status should be monitored to individualize iron supplementation of preterm neonates [14].

In case of IDA in preterm newborns, elemental enteral iron supplementation is increased to 3–6 mg/kg per day for three months [93].

The current recommendation may not be adequate to meet the needs of all premature infants. The most immature neonates, such as ELBW, have a poor iron endowment and may require extra iron due to the catch-up growth and increased erythropoiesis. Indeed, 15% of infants with birth-weight <1301 g are iron deficient at two months of age even if supplemented with 4–6 mg/kg of iron since the second week of age [95]. Nevertheless, the majority of ELBW infants receive 3–5 RBC transfusions during their hospital stay that improve their iron stores [16]. For these reasons, the iron status of ELBW infants should be monitored to tailor iron administration.

No gastrointestinal adverse effects have been described after the administration of iron-rich formula or elemental iron. Hematochezia has been reported in 17% of premature infants exposed to high doses of medical iron (8–16 mg/kg/day) [90]. However, a causal link with oral iron administration was not established, and iron supplementation can be resumed after the resolution of symptoms [16].

Concerns were raised regarding the possible interaction in absorption between iron and other divalent cations since they share the same gut transport mechanism (divalent metal transporter 1, DMT1). However, iron supplementation at the recommended doses does not interfere with zinc or selenium uptake, and zinc supplementation does not compromise iron absorption [16,96]. Conversely, it is known that oral iron alters copper metabolism, although further research is needed [97].

Iron supplementation may induce oxidative stress by promoting ROS production as a result of increased intra- and extra-cellular free iron concentrations [98], whose underlying mechanism has been previously mentioned (cfr paragraph 2, Figure 1). 

#### 4.3.2. Parenteral Iron Supplementation

In addition to the oral route, iron can be administered parenterally. Intramuscular administration is not recommended because it is painful and prone to complications, while intravenous (i.v.) iron appears to be safe [99]. Although the daily iron intake during the third trimester is about 1.6–2 mg/kg/day, a dose of 120 µg/kg/day is adequate and, since there is no physiologic regulatory mechanism for iron excretion, almost the totality of it gets stored in tissues [16]. When compared with the enteral route, i.v. supplementation is associated with higher SF levels, while inconclusive results are related to its efficacy in supporting erythropoiesis [99]. Besides, the need for i.v. line in a full enterally-fed infant makes the i.v. route unreasonable in clinical practice. Moreover, a transient rise of malondialdehyde (MDA), indicating lipid peroxidation, has been reported after iron infusions, thus suggesting a risk of oxidative stress [16]

### 4.4. Risk Groups Requiring Tailored Iron Supplementation

Preterm infants treated with rHuEPO require higher doses of iron because rHuEPO improves growth, stimulates erythropoiesis, and decreases the number of RBC transfusions at the expense of tissues iron stores, as reflected by the decrease in SF after the initiation of rHuEPO therapy [90,100]. The AAP recommends an oral iron supplementation of 6 mg/kg/day during rHuEPO therapy [93]. This dose is appropriate to sustain erythropoiesis; however, it may be inadequate to preserve body iron stores. No differences have been found in the SF levels nor in the hematological response after an oral iron supplementation at a high dose (16 mg/kg/day) or low dose (8 mg/kg/day) in infants treated with rHuEPO, as reported by Bader et al. [90].

Ferritin concentration higher than 100 µg/L should be considered the threshold to guide iron administration during rHuEPO therapy [16].

Despite the lack of specific recommendations, infants with increased SF concentrations (>350 µg/L) might require personalized iron supplementation. This condition can indicate two different states:

1. Iron overload (e.g., after recurrent erythrocyte transfusions); or
2. Iron sequestration (e.g., in the setting of an inflammatory process).

While the former group is at risk of iron overload associated insults, and should not be supplemented, the latter can suffer from bone marrow ID and subsequent insufficient erythropoiesis and thus could benefit from iron administration. Assessing bone marrow iron status by measuring reticulocyte count and ZnPP/H ratio can support decision making [16].

Nevertheless, one out of four premature neonates with SF levels >95th percentile at hospital discharge will manifest ID at 6–12 months of age if iron supplementation is discontinued [16].

### 4.5. Timing of Iron Supplementation and Screening of ID

Iron supplementation should start from four to six weeks of age [16] when serum iron and ferritin concentration start reducing and iron incorporation in RBC occurs more efficiently [16]. The AAP does not recommend iron supplementation before the second week of age, due to the immature antioxidant capacity before that age [4]. In contrast, iron supplementation, by two weeks of age, as suggested by ESPGHAN [94], is associated with decreased rates of RBC transfusions and incidence of ID at 2–6 months of age when compared with later onset. Only one study evaluated the long-term effect of early (started at a median age of 14 days) versus late (eight weeks) iron supplementation reporting better cognitive and motor outcomes at five years of age following the earlier start [41]. However, this study was underpowered to evaluate improvements in neurocognitive development [41,101]. Therefore, in the absence of consistent data, considering the potential risk of iron administration in the first month of life due to iron overload and immaturity of the gastrointestinal function, caution is required, especially in ELBW infants.

Iron supplementation should be provided at least up to six months of age when weaning with iron-rich foods begins [94]. The AAP recommends prolonging iron supplementation until the end of the first year of life [93].

The iron status of premature infants should be monitored periodically. The frequency and timing of follow up controls should be scheduled, taking into account the iron status at discharge, iron supplementation, and type of feeding [16].

## 5. Iron Overload and Toxicity

### 5.1. Risk Factors

#### 5.1.1. RBC Transfusions

Transfusion-derived iron is the main cause of iron overload in premature infants. Around 80% of VLBW and 95% of ELBW infants require at least one RBC transfusion during hospitalization, with 0.5–1 mg of iron intake for each mL of packed RBC transfused. [16].

Biologically, the exceeding iron cannot be actively excreted by humans. Consequently, after multiple transfusions, higher serum and ferritin concentrations and liver iron storage can be found in preterm infants [16].

The pathogenesis of prematurity-related comorbidities is multifactorial. The association between the rate of erythrocyte transfusions and the incidence of NEC, IVH, ROP, and BPD has been suggested [21,102,103,104]. In this context, iron (excess) has been hypothesized to play a causative role due to its impact on the immune system [105], nitric oxide-induced vasoregulation [106], and oxidative stress [107]. Specifically, both the preparation and storage of pediatric packed RBCs may predispose to redox unbalance. Indeed, pediatric RBCs are prepared from adult blood, by replacing most of the plasma with additive solutions, thus reducing the net amount of iron-binding proteins and antioxidants [108].

During storage, the exposure of blood to shear stress, plastic bags, anticoagulants, and additives contributes to the increase of extracellular iron and NTBI [109], leading to a rise in MDA [110].

In adults, the storage duration of transfused RBCs does not impact on mortality [111]. In pediatrics, there is a low level of evidence stemming from observational studies of the association between longer storage duration of transfused RBCs and worse outcomes [112]. However, data from clinical trials do not support the use of fresh blood [113,114]. Donor exposure is another relevant issue, especially when it comes to the smallest and sickest premature infants, requiring multiple transfusions in the early days of life. The use of satellite bags (small-volume aliquots from the same unit of donor blood) may limit the donor exposure rate [115,116].

Finally, procedures that require massive blood products exposure, as in the case of exchange transfusion or extracorporeal membrane oxygenation, further increases the neonatal exposure to hemolysis, oxidative stress and, hence, the risk of mortality [117,118].

#### 5.1.2. Excessive Iron Supplementation

At routinely-used dosage, parenteral or enteral iron does not produce oxidative stress injury, even if a transient peak of serum iron level can be registered after administration [16]. It is reported that even an enteral supplementation at higher doses (up to 18 mg/kg/day) [119] does not cause oxidative stress damage but, instead, stimulate erythropoiesis, as low ZnPP/H ratio testify [66]. In VLBW infants, a one-week course of high doses of oral iron (18 mg/kg/day) did not result in a change in oxidative stress biomarkers nor anti-oxidants levels [89].

However, lower doses (3–6 mg/kg/day) for a longer period (up to nine months of age) produce an increase in glutathione peroxidase concentrations, a marker of oxidative stress [120]. Moreover, large doses of enteral iron administration have been linked to hemolysis in preterm infants with vitamin E deficiency [16].

### 5.2. Iron Toxicity

Iron excess has been associated with poor growth and interference with zinc and copper metabolism [26]. The relationship between iron and infection is widely recognized [121]. Previous studies reported an increased incidence of respiratory tract infections in neonates supplemented with high iron doses (formula fortified with 20.7 mg iron/L) [120]. Certainly, NTBI acts as a potent pro-oxidant agent through the generation of ROS.

Recently, a trial showed that iron-fortified foods modulate the intestinal microbiota, with overgrowth of pathogenic enterobacteria, thus triggering gut inflammation [122]. The consequences of this process are not yet known and deserve to be explored.

Premature infants are vulnerable to iron toxicity due to low TIBC levels and immature anti-oxidant defenses that facilitates iron oxidation of the ferrous state with the increase of ROS production [16]. Vitamin E and vitamin C are radical-scavenging antioxidants, which are poorly adsorbed until the 34th week of gestation and low activity in the first two weeks of life [4]. Finally, superoxide dismutase (SOD), which is an enzyme with a key antioxidant role, is lacking in preterm neonates [4,25].

A recent study involving 95 premature neonates found no association between moderate iron overload, as defined by SF >400 ng/mL but <1000 ng/mL at 34–35 weeks of corrected age and neurodevelopmental impairment at 8–12 months [119]. However, preterm newborns in the first weeks of life are prone to develop oxidative stress, due to concomitant predisposing risk factors. Therefore even modest SF levels may damage the brain [119].

The early identification of biomarkers of oxidative stress could prevent future sequelae and allow new therapeutic strategies. To date, serum and urinary prostanoids, particularly urinary 8-isoprostane, and visfatin, an adipocytokine involved in inflammation, have been identified as potential markers of oxidative stress in premature newborns [25,89,123].

Additionally, the dosage of plasma antioxidants, such as glutathione, vitamin E, and vitamin C (ascorbic acid), particularly oxidized to total ascorbic acid ratio (DHAA/TAA), has been proposed to prevent oxidative stress [89]. Recently, serum gamma-glutamyltransferase (GGT) has been found to have a role in ROS production and glutathione synthesis, and it might be used as a cheap and reliable marker of oxidative stress [124]. Plasma adenosine appears as a promising biomarker to predict brain damage; however, its role in the neonatal setting has yet to be demonstrated [25].

## 6. Concluding Remarks and Future Perspectives

Many preventive measures have already been implemented in clinical practice to tackle ID, and iron supplementation is recommended for all preterm neonates. However, since iron levels are influenced by several perinatal factors, a tailored-supplementation based on laboratory iron status parameters should be encouraged.

For this reason, gestational age-related reference ranges for the main iron-related diagnostic indices should be established, especially for the most immature neonates. Attention should be paid to the new ID markers, such as RET-He and CHr, which could anticipate ID diagnosis, thus allowing a prompt therapy.

Beyond ID and IDA, which have been widely addressed, iron overload is emerging as a ‘new’ issue in the management of sick preterm infants. Future efforts should focus on the early identification of biomarkers of oxidative stress, which could improve patients’ care by paving the way for innovative therapeutic targets.

**Figure 2:**
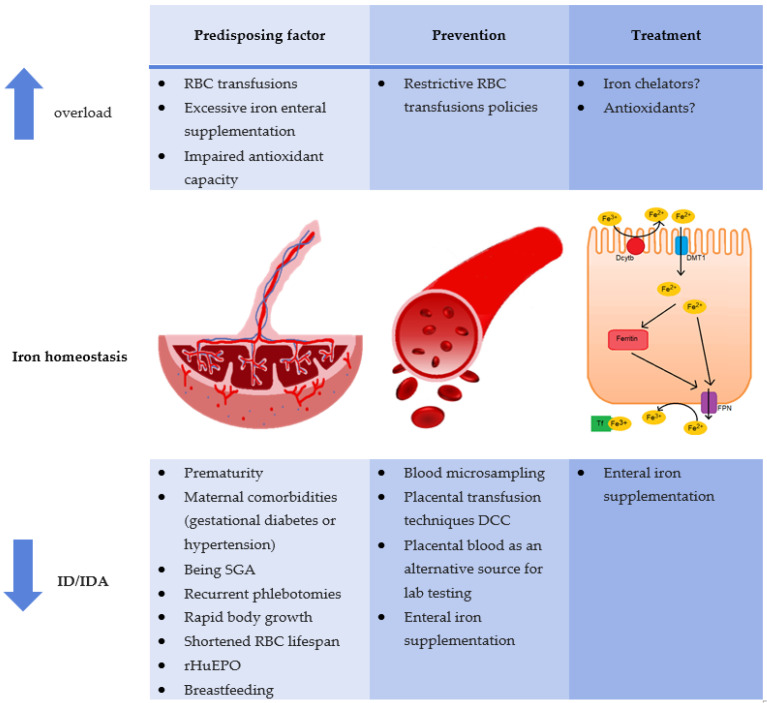
Iron homeostasis in preterm newborns: risk factors, prevention strategies and treatment.

The main risk factors, prevention strategies, and therapies for iron deficiency and overload are summarized in Figure 2.

DCC: delayed cord clamping; Dcytb: duodenal cytochrome b; DMT1: divalent metal transporter; Fe^2+^: ferrous cation; Fe^3+^: ferric cation; FPN: ferroportin; RBC: red blood cell; rHuEPO: recombinant human erythropoietin; SGA: small for gestational age; TF: transferrin.

## Figures and Tables

**Table 1:** Iron status parameters: their response to ID, IDA and iron overload [14,54,55].

Parameter	ID	IDA	Iron Overload
Hb	Normal	Reduced	Normal
MCV	Normal	Reduced	Normal
RET-He/CHr	Reduced	Reduced	Normal
SF	Reduced	Reduced	Increased
Transferrin saturation	Reduced	Reduced	Increased
sTfR1	Increased	Increased	Reduced
ZnPP/H ratio	Increased	Increased	Reduced

ID, iron deficiency; IDA, iron deficiency anemia; Hb, hemoglobin; MCV, mean corpuscular volume; RET-HE, reticulocyte hemoglobin equivalent; CHr, mean cellular hemoglobin content of reticulocytes; SF, serum ferritin; sTfR1, serum transferrin receptor; ZnPP/He ratio, zinc protoporphyrin to heme ratio.

**Table 2:** Reference ranges for the main iron status parameters in term and preterm neonates.

	Cord Blood		Capillary Blood (within 72 h from Birth)		
	Preterm	Term	Preterm	Term	
Hb (g/dL)	12.4–19.2	13.3–18.4		14.5–22.5	Lorenz et al. 2013 [57]
MCV (fL)	103–133	97.8–118.5		95–121	Lorenz et al. 2013 [57]
Serum ferritin (µg/L)	35–267	40–309			Siddappa et al. 2007 [28]
STfR1 (mg/L)	6.1–13.7	6.4–10.6			Sweet e al. 2001 [58]
ZnPP/H ratio (µmol/mol)	55–135.5	49.6–108.4			Juul et al. 2003 [59]
RET-He (pg)		27.4–36	24.3–36.2	25.5–37.6	Löfving et al. 2018 [60] Lorenz et al. 2017 [61]

Hb, hemoglobin; MCV, mean corpuscular volume; sTfR1, serum transferrin receptor; ZnPP/He ratio, zinc protoporphyrin to heme ratio; RET-HE, reticulocyte hemoglobin equivalent. All are central 95% reference intervals, except for SF that is central 90% and STfR1 that is the interquartile range.

## List of Abbreviation

*AAP*	American Academy of Pediatrics
*ABR*	Auditory brainstem-evoked response
*AGA*	Appropriate-for-gestational-age
*BPD*	Bronchopulmonary dysplasia
*CHr*	Reticulocyte hemoglobin content
*CRP*	C reactive protein
*CSF*	Cerebrospinal fluid
*DCC*	Delayed cord clamping
*DHAA/TAA*	Oxidized to total ascorbic acid ratio
*DMT1*	Divalent metal transporter 1
*ELBW*	Extremely low birth weight
*ESPGHAN*	The European Society for Pediatric Gastroenterology, Hepatology, and Nutrition
*GGT*	Gamma-glutamyltransferase
*Hb*	Hemoglobin
*HIE*	Hypoxic-ischemic encephalopathy
*ICH*	Intracerebral hemorrhage
*ID*	Iron deficiency
*IDA*	Iron deficiency anemia
*IUGR*	Intrauterine growth restriction
*i.v.*	Intravenous
*IVH*	Intraventricular hemorrhage
*MDA*	Malondialdehyde
*MCV*	Mean corpuscular volume
*NEC*	Necrotizing enterocolitis
*NICU*	Neonatal Intensive Care Unit
*NTBI*	Non-transferrin-bound iron
*PVL*	Periventricular leukomalacia
*PWM*	Punctate white matter lesions
*RBC*	Red blood cells
*RET-He*	Reticulocyte hemoglobin equivalent
*rHuEPO*	Recombinant human erythropoietin
*ROP*	Retinopathy of prematurity
*ROS*	Reactive oxygen species
*SF*	Serum ferritin
*SGA*	Small-for-gestational-age
*STfR1*	Soluble transferrin receptor
*TACO*	Transfusion-associated circulatory overload
*TfR1*	Transferrin receptor
*TIBC*	Total iron bind capacity
*TRALI*	Transfusion-related acute lung injury
*UCM*	Umbilical cord milking
*VLBW*	Very low birth weight
*ZnPP/H*	Zinc protoporphyrin to heme ratio
